# The British Journals, &c.: Anatomy and Physiology

**Published:** 1842-07

**Authors:** 


### II. THE BRITISH JOURNALS.

### ANATOMY AND PHYSIOLOGY.

Congenital Luxation of the Inferior Maxilla. An idiot, thirty-eight years of age, died of a pulmonary affection, the particulars of which, having no relation to the object of the paper, are not given. On entering the room where the body was lying, the author's attention was arrested by a curious deformity of the countenance, a representation of which, along with various woodcuts, illustrative of the abnormal development of the facial bones, is given in the original article. The right and left sides of the face seemed as if they did not belong to the same individual; the left being in every respect larger and more fully developed than the other. The muscles of the right side, as the masseter, pterygoid, and temporal muscles appeared small in comparison with their fellows of the opposite side, yet as regarded colour and consistence, perfectly healthy. The external lateral ligament of the lower jaw, instead of taking its natural direction downwards and backwards, was seen descending obliquely forward, to be fixed into an imperfectly developed condyle, which was not in contact with the articulating portion of the temporal bone, but was separated from it by an interval of at least a quarter of an inch. There was no inter-articular cartilage, nor cartilage of incrustation, the osseous surfaces of the joint being invested by a thick periosteum alone. The bones in general of the right side of the face were smaller than the corresponding ones of the left. The right half of the inferior maxillary bone was considerably smaller than the left, the atrophy extending forwards as far as the mental foramen, and affecting the bone in its length, breadth, and thickness; the parotideal margin was thin, and terminated above in a small curved process, directed nearly horizontally inwards and upwards. This process, which somewhat resembled in form the coracoid process of the scapula, was the only trace of a condyle, and was destitute of cartilage. There was, in short, a complete arrest of the condyle.—The *temporal bone:*—The superior longitudinal root existed, but the inferior transverse root, or the articular eminence, was not developed, there being in its place merely a flat surface destitute of cartilage. The transverse root of the zygoma not being developed, there was consequently no glenoid cavity. The right malar bone was smaller than its fellow; its zygomatic process of extraordinary length extended back as far as the tubercle of the zygoma, thus forming the entire of the zygomatic arch, which was concave externally, and convex towards the zygomatic fossa, the reverse of what obtains in the normal state. The superior maxillary bone was much smaller than its fellow; the palate did not consist of two symmetrical portions, the suture being directed from before and from the right, backwards to the left side. The motions of which the lower jaws were capable, were much more extensive than the normal ones, especially lateral motions.

It is obvious that all the other deviations, from the normal state of the bones, were consequent on the arrest of development in the transverse root of the zygoma, since it is, in fact, by the growth of this process, that the glenoid cavity is formed, and therefore that the absence of the condyle of the jaw, the atrophy of its ramus, of the superior maxillary and malar bone were consequent on and to be referred to the orignal malformation of the glenoid portion of the temporo-maxillary articulation. The resemblance of this form of the articulation to that of the rodentia must be apparent to the comparative anatomist. No analogous malformation has been recorded; it is therefore an addition to our knowledge of congenital luxation, and another instance of original articular malformation coexisting with idiotcy. Robert W. Smith, Esq., *Dublin Journal of Medical Science*, No. 62, *May*, 1842.

Functions of the Spinal Cord in Cold-blooded Animals. The object of the author of this paper is to prove that we are not justified in attributing to the reflex function of the spinal cord, many of those movements which are seen in frogs and other cold-blooded animals after decapitation. We cannot but consider, however, that his arguments are insufficient. In the first place he states it as an established fact that no cineritious matter has been detected in the spinal cord of frogs and other reptiles. We are not aware of the authority on which this assertion rests; and we have no hesitation in saying that it is virtually untrue, for though no nervous matter having the cineritious *colour* may be present there, yet a substance having a *structural* correspondence with that which is found in the spinal cord of warm-blooded animals undoubtedly exists there. Dr. Paton adduces many experiments which appear to him to prove that not only sensation but perception and volition remain in the spinal cord of frogs after the removal of the brain and medulla oblongata,—a position in which we apprehend that few will concur with him. His arguments are based solely on the *adaptiveness* of the movements performed by the animals, which adaptiveness, being equally great in actions which are universally acknowledged to be automatic, cannot be relied on as a test. There is no doubt that it is more remarkable in the cold-blooded vertebrata than it is in the higher classes; but this corresponds with the general fact that the influence of the brain on the motor acts diminishes as we descend the scale, and at last almost ceases. Among the invertebrata we find the most perfectly *adaptive* actions performed by ganglia, in which volition cannot be imagined to exist, and in which we have no reason to suppose that sensibility resides. Is it to be imagined, for example, that when the nervous column of a centipede has been cut into several divisions, each becomes a distinct centre of consciousness and will,—an independent *ego*,—because the legs move when in contact with a hard surface? Or to go to the vegetable kingdom, is the perfect adaptiveness of the movements of the dionœa to be taken as an indication of *its* consciousness and voluntary power? Without enlarged comprehensive views on this subject, we do not think that right conclusions are likely to be attained.—Dr. PATON, *Edinburgh Medical and Surgical Journal, April,* 1842.

---

PHYSIOLOGY OF THE SALIVA. The following is the summary of a series of papers on the physiological uses of the saliva:

*Active uses.* 1. To stimulate the stomach and excite it to activity by contact. 2. To aid the digestion of food by a specific action upon the food itself. [The author here adds in note, that during the act of assisting the digestion of food, *the saliva is itself digested.*] 3. To neutralize any undue acidity in the stomach by supplying a proportionate alkali.

*Passive uses.* 1. To assist the sense of taste. 2. To favour the expression of the voice. 3. To clear the mucous membrane of the mouth, and to moderate thirst.—Dr. WRIGHT, *Lancet,* No. viii. *May* 21, 1842.

---

NORMAL DIMENSIONS OF THE HEART IN THE ADULT. The author gives an account of the plan of measurement which he adopted, which seems to have been one calculated to ensure accuracy: of upwards of 100 hearts, of which the dimensions were taken, care was had to exclude every one which exhibited any trace of organic change. We shall only give the *mean* measurements.

Of 15 male hearts, the mean *circumference* was 9 inches 27-48ths; of 17 female hearts, 8 inches 16-48ths.

The mean *length* of the male heart was 4 inches 16-48ths; of the female, 4 inches 36-48ths.

The mean *thickness* of the *left* ventricle in the male was 27-48ths of an inch; in the female, 23-48ths; of the *right* ventricle, in the male, 8-48ths of an inch; in the female, 6-48ths.

The septum ventriculorum has, in the male, a mean thickness of 22-48ths of an inch; in the female, 14-48ths.

The aortic orifice in the male has a mean circumference of 2 inches 31-48ths; in the female, 2 inches 22-48ths.

In the male, the pulmonary artery at its origin has a mean circumference of 2 inches 34-48ths; the right auriculo-ventricular orifice, 4 inches 35-48ths; the left auriculo-ventricular orifice, 3 inches 45-48ths; and the corresponding parts in the female are relatively less.

The following are the author's deductions: 1st. The male is larger than the female heart. 2d. The length of the healthy heart to its circumference is rather less than 1 to 2. 3d. The thickness of the right ventricular parietes to the left is as 1 to 3, nearly. 4th. The pulmonary artery is slightly wider than the aorta. 5th. The right auriculo-ventricular orifice is considerably larger than the left.

Dr. RANKING, *Medical Gazette*, No. xxiv. 1842.

---

CAN THE MALE INFLUENCE THE DURATION OF THE FŒTUS IN UTERO? The Earl of Spencer has observed that cows in calf to a particular bull belonging to his lordship carry their calves about four days longer than cows in calf to any other bull, The average period of gestation of the cow his lordship finds to be 248 to 285 days; but cows in calf to the bull referred to do not bring forth till 290¼ days. His lordship has therefore proposed the query, whether the bull can exert any influence on the duration of the calf in the uterus of its mother, and whether there be any work extant in which this question is handled?—Dr. HALL, *Medical Gazette*, No. xxiii. *May* 6, 1842.

